# Botryosphaeriaceae Species Causing Stem Blight and Dieback of Blueberries in Serbia

**DOI:** 10.3390/jof11090686

**Published:** 2025-09-22

**Authors:** Miloš Marić, Mira Vojvodić, Darko Jevremović, Bojana Vasilijević, Tanja Vasić, Miljan Grkinić, Aleksandra Bulajić

**Affiliations:** 1 Field Test doo, 11000 Belgrade, Serbia; milos.maric.somi@gmail.com; 2 Department of Phytopathology, Institute of Phytomedicine, Faculty of Agriculture, University of Belgrad, 11080 Belgrade, Serbia; miravojvodic2510@gmail.com (M.V.); beomiljan@gmail.com (M.G.); 3 Fruit Research Institute, 32000 Čačak, Serbia; darkoj@ftn.kg.ac.rs (D.J.); bvasilijevic@institut-cacak.org (B.V.); 4 Faculty of Agriculture Kruševac, University of Niš, 37000 Kruševac, Serbia; tanjavasic82@gmail.com

**Keywords:** diagnosis, morphology, phylogeny, cultivar susceptibility

## Abstract

In the main growing areas in Serbia, plants with symptoms of stem blight were sampled in nine orchards with American highbush blueberry (*Vaccinium corymbosum*), cultivar ‘Duke’, with high disease incidence, and 153 samples were taken. A total of 128 Botryosphaeriaceae isolates were characterized on the basis of morphology, sequence analysis, multilocus phylogeny based on ITS, *TEF1-α* and *TUB2* sequences and pathogenicity, and belonged to one of the four species *Neofusicoccum parvum*, *Botryosphaeria dothidea*, *Diplodia seriata* and *Lasiodiplodia iraniensis*. Both *D. seriata* and *L. iraniensis* were detected for the first time on blueberries in Serbia, and *L. iraniensis* was detected for the first time on blueberries worldwide. Comparative morphological and *TEF1-α* sequence analyses allowed a clear separation of *L. iraniensis* from the phylogenetically closely related *L. fujianensis*, *L. thailandica* and *L. endophytica*. Of the nine blueberry cultivars ‘Aurora’, ‘Barbara Ann’, ‘Bluecrop’, ‘Bluejay’, ‘Draper’, ‘Duke’, ‘Huron’, ‘Patriot’ and ‘Spartan’ inoculated with *L. iraniensis* (isolate 421-19), the cultivar ‘Duke’ was the most susceptible. In our study, the majority of orchards were in their second or third year of production, implying that the planting material is likely to be the source of infection, emphasizing the importance of pathogen-free planting material.

## 1. Introduction

The American highbush blueberry (*Vaccinium corymbosum* L., Fam. Ericaceae) is commercially cultivated worldwide under various climatic conditions [1] as its fruits have exceptional nutritional properties and positive effects on health [2]. As it is a very profitable crop, the global production of blueberries is constantly increasing, with more than 1.2 million tonnes being produced in 2022, and America being the largest producer (979,668 tonnes), followed by Europe (207,915 tonnes) (https://www.fao.org/faostat, accessed on 15 May 2025). In Serbia, the production of highbush blueberries is also increasing very rapidly, much faster than the production of any other fruit species [3] and is currently grown on over 2500 ha [4].

Blueberry production worldwide can be affected by a number of biotic and abiotic factors, and among these, fungi from the Botryosphaeriaceae family are considered one of the most important and devastating factors limiting blueberry production [5,6,7,8,9]. In New Zealand, diseases caused by Botryosphaeriaceae are considered particularly devastating in newly planted orchards, where nearly 20% of plants are infected and significant annual costs are incurred due to yield loss and replanting costs [6]. The most important factor contributing to the rapid spread of Botryosphaeriaceae disease is probably related to the health status of the planting material [10]. Due to the broad host range, latent infections, the ability to infect plants via wounds and the limited possibilities of efficient disease control [11], Botryosphaeriaceae pose a major challenge to the production of numerous host plants, including blueberries. The fungal family Botryosphaeriaceae currently comprises 24 defined genera with diverse lifestyles, saprobes, endophytes and pathogens associated with a wide range of host plants. Among the Botryosphaeriaceae, the genera *Botryosphaeria*, *Diplodia*, *Lasiodiplodia*, *Neofusicoccum*, *Dothiorella* and *Neoscytalidium* are the most important and best-studied plant pathogens [12].

Although previously considered a possible synonym of *Diplodia* [13], the fungal genus *Lasiodiplodia* Ellis & Everh has long been recognized and is well defined based on the morphology of the pycnidia, the longitudinal striation of the mature conidia and phylogenetic studies [14,15]. *Lasiodiplodia* is a very dynamic genus with over 47 established species to date, with new species being described relatively frequently [16,17]. Some *Lasiodiplodia* species are even considered to be of quarantine importance, such as *L. pseudotheobromae* [18] and more recently, *L. iraniensis* [19]. *Lasiodiplodia iraniensis* is a relatively newly described species that occurs as a pathogen of *Salvadora persica*, *Juglans* spp., mango, *Eucalyptus* spp., *Citrus* spp. and tropical almonds in Iran [20]. Several studies have shown that the status of isolates identified as *L. jatrophicola* as a closely related but distinct species from *L. iraniensis* is not justified, and it is currently synonymized with *L. iraniensis* [19,21,22,23]. After the initial description, *L. iraniensis* was found on mango in Western Australia [24], the United Arab Emirates [25], Brazil [26] and Peru [22], on mandarins in the United Arab Emirates [25], *Bougainvillea spectabilis* in southern China [27], *Anacardium occidentale* in Brazil [28], Persian lime in Mexico [23] and, more recently, on *Adansonia digitata* in Mozambique [29], *Eucaliptus* in India [30], bananas in Brazil [31] and yam and sweet oranges [19,32] in the USA. In all these regions and on all host plants, *L. iraniensis* has been described as an aggressive and economically important pathogen.

The intensive increase in blueberry production in Serbia has been accompanied by the appearance of various symptoms of stem blight of a largely unknown origin, which has triggered research that has recently confirmed the presence of blueberry strain pathogens including *Macrophomina phaseolina* [33], *Fusarium sporotrichioides* [34], *Neopestalotiopsis clavispora* [35] and *N. vaccinii*, *N. rosae*, *Diaporthe eres*, *D. foeniculina* and *Neofusicoccum parvum* [36]. During the study of blueberry stem diseases, we obtained a considerable number of Botryosphaeriaceae isolates from symptomatic plants, and our main objectives were as follows: (i) to identify the causal pathogens; (ii) to investigate morphological characteristics and the ability to grow at extreme temperatures; (iii) to determine the taxonomic position of the obtained isolates based on the sequences of ITS rDNA, translation elongation factor 1α (*TEF1-α*) and *β*-tubulin (*TUB2*); (iv) to determine the phylogenetic relationship between the Botryosphaeriaceae and, in particular, between the *Lasiodiplodia* spp. isolates and the relationship with newly detected species in Serbia; and (v) to evaluate the susceptibility of nine blueberry cultivars grown in Serbia and worldwide to selected discovered *Lasiodiplodia* sp.

## 2. Materials and Methods

### 2.1. Sampling and Isolations

The blueberry stem disease survey was conducted from 2011 to 2022, and the field inspections covered nine production fields/locations in four administrative districts in Serbia. The blueberry cultivar ‘Duke’ was grown in all orchards and a total of 153 samples were collected (Table 1). Disease incidence was calculated in each orchard (by zigzag inspections and random assessment of 100 plants in three replicates), and 5–25 samples were collected, depending on size of the orchard and symptoms. Small woody fragments of necrotic tissue were taken from each sample, surface sterilized with 2% sodium hypochlorite, placed on potato dextrose agar (PDA; 200 g potato, 20 g dextrose, 17 g agar and 1 litre distilled H_2_O) [37] and incubated at 24 °C for 5 days. One or more representative colonies with the same morphology were selected from each of the nine growing fields, from which monosporial isolates were obtained for further characterization. The isolates were stored on sealed PDA slants at 4 °C in the fungal collection of the Department of Phytopathology, Institute of Phytomedicine, University of Belgrade—Faculty of Agriculture.

**Figure 1:**
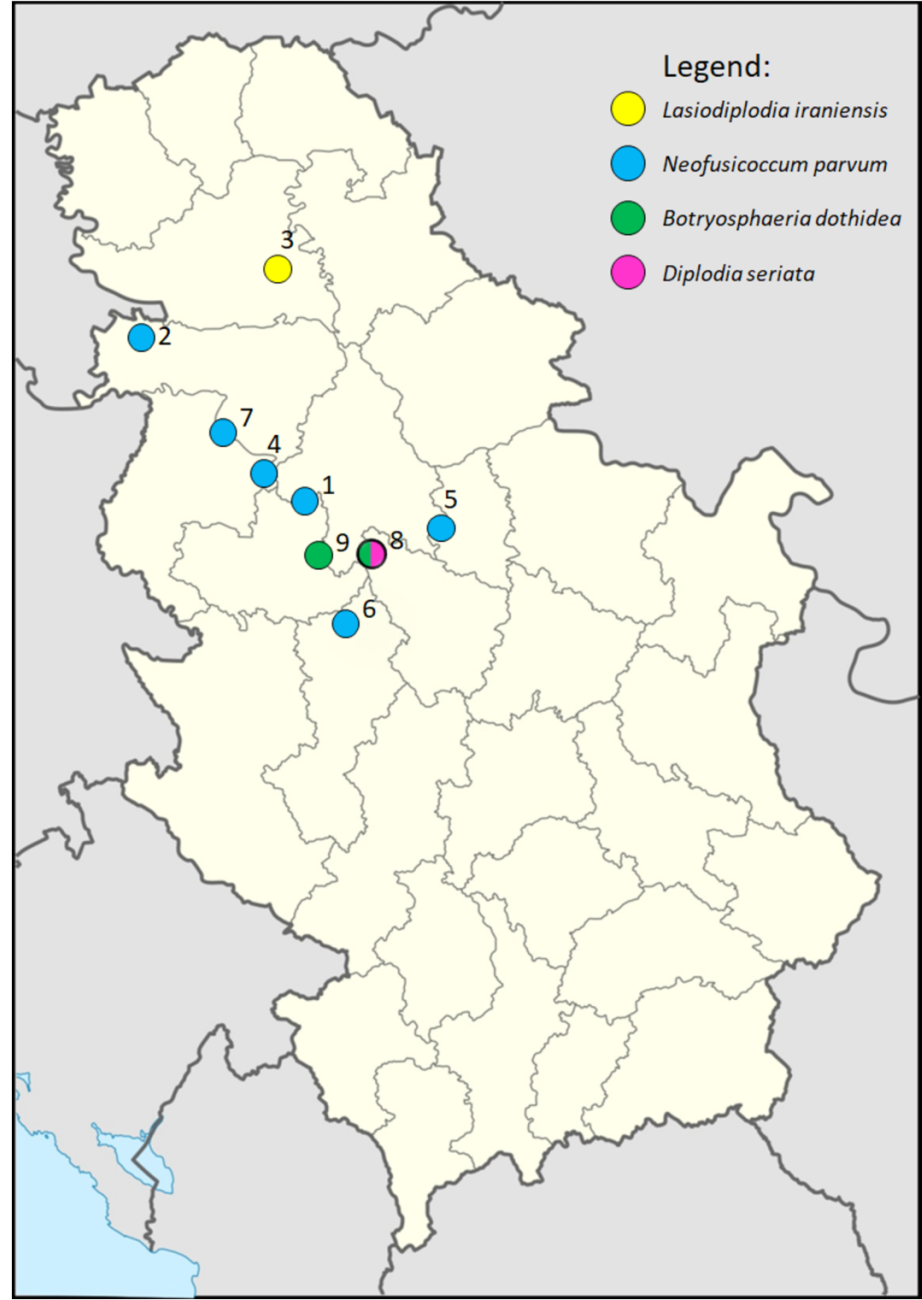
Geographic distribution of localities in Serbia included in the survey and detected isolates.

### 2.2. Morphological and Ecological Characterization

Colony appearance, including colour and shape, was assessed 14 dpi (days post inoculation) on PDA at 24 °C in the dark. Growth rate was determined by measuring two perpendicular colony diameters in five replicates per isolate and calculating an average value for each isolate. To induce sporulation, isolates were cultured on pine needle agar (PNA: 17 g agar, 1 litre distilled H_2_O and sterilized pine needles placed onto the medium) [38]. The presence and appearance of pycnidia and conidia were observed at 14, 21, 28 and 35 dpi using a compound microscope (Olympus CX41, Olympus Europa SE & Co. KG), and the dimensions of pycnidia and immature and mature conidia were measured (50 and 100 randomly selected, respectively). The selected *Lasiodiplodia* spp. isolates were physiologically characterized based on colony appearance and ability to grow on PDA at temperatures of 5, 10, 15, 25, 35, 37.5 and 40 °C, as determined by measuring two perpendicular colony diameters in five replicates per isolate and calculating an average value for each temperature. Data were analyzed with SPSS (version 29, IBM, NY, USA) using one-way ANOVA followed by Duncan’s multiple range test at *p* < 0.05.

### 2.3. DNA Amplification and Sequencing

Total genomic DNA was extracted using the DNeasy Plant Mini Kit (Qiagen, Hilden, Germany) from 100 mg of dry mycelium from 7-day-old cultures of 38 selected isolates grown in potato dextrose broth (PDB; 200 g potato, 20 g dextrose and 1 L distilled H_2_O), following the manufacturer’s instructions. PCR amplification of three genomic regions, including ITS rDNA (38 isolates), *TEF1-α* (24 isolates) and *TUB2* (24 isolates), was performed using the primers ITS1F/ITS4 [39,40], Bt2A/Bt2B [41] and EF1-728/EF1-986 [42], on annealing temperatures 52 °C, 55 °C and 58 °C, respectively. All reactions were performed in a total volume of 25 μL consisting of 12.5 μL of 2× PCR Master Mix (Fermentas, Lithuania), 6.5 μL of RNase-free water, 2.5 μL of both forward and reverse primers (working solution with a final concentration of 100 pmol/μL, Metabion International, Germany) and 1 μL of template DNA. The amplification conditions were as follows: initial denaturation at 94 °C for 5 min, followed by 40 cycles of denaturation at 94 °C for 30 s, variable recommended annealing conditions, elongation at 72 °C for 1 min and final elongation for 10 min at 72 °C. The amplicons obtained were stained with ethidium bromide, analyzed by 1% agarose gel electrophoresis and visualized with a UV transilluminator. The PCR products of all genomic regions were sequenced directly in both directions with an automatic sequencer (Automatic Sequencer Macrogen Inc., The Netherlands) using the same primers as for amplification. The consensus sequences were calculated with ClustalW [43], integrated into the software MEGA X [44], and deposited in GenBank (http://www.ncbi.nlm.nih.gov, accessed on 1 April 2025).

### 2.4. Sequence and Phylogenetic Analyses

Sequences generated from the selected 38 isolates were compared with each other by calculating nucleotide (nt) similarities, as well as with previously deposited isolates available in the GenBank, using the similarity search tool BLAST (version 2.13.0, NCBI) for identification at the genus level.

Multilocus phylogenetic sequence analyses (ITS rDNA, *TEF1-α* and *TUB2*) were performed on two data sets, one to clarify the position of 24 Serbian isolates within the family Botryosphaeriaceae and the other to clarify the position of five *Lasiodiplodia* isolates within the genus *Lasiodiplodia*. The targeted analyses of Botryosphaeriaceae included 10 previously listed type-derived species (38 reference isolates) and *Melanops tulasnei* [45,46,47], while other targeted analyses included 34 previously listed type-derived species (49 reference isolates) and *Dothiorella viticola* [48] as an outgroup (Table 2) with gaps and missing data treated as missing characters. The phylogenetic trees were inferred using the Maximum Likelihood method implemented in MEGA X software [44]. Gamma distributed Tamura-Nei model (G+I) determined by a Model test implemented in MEGA X was used as the best-fitting model of nucleotide substitution. All sites with gaps were omitted. The reliability of the obtained trees was evaluated with 1000 bootstrap replicates.

The position of the Serbian *Lasiodiplodia* isolates was further evaluated based on the nucleotide polymorphism within the *TEF1-α* gene. A total of 19 sequences of the closely related *L. iraniensis*, *L. fujianensis*, *L. thailandica* and *L. endophytica* were aligned and analyzed using the sequence of *L. endophytica* as a representative [49].

### 2.5. Pathogenicity Testing

The pathogenicity of 70 Botryosphaeriaceae isolates (40 *N. parvum*, 20 *B. dothidea*, 5 *D. seriata* and 5 *L. iraniensis*) was tested by the artificial wound inoculation of branches of a 6-year-old healthy blueberry ‘Duke’ from a collection orchard of the Fruit Research Institute Čačak, Serbia, using mycelial plugs, as previously described [16,30,50]. Well-developed, symptomless blueberry branches were superficially sterilized and a clear cut approximately 0.5 cm long incision was made with a sterile scalpel blade without damaging the underlying cambial tissue. Mycelial plugs (5 mm diameter) from the edge of a 4-day-old PDA culture grown at 24 °C were placed under the bark (mycelial surface facing downwards) and the wound was sealed with sterilized moist cotton wool and Parafilm. As a negative control, branches were inoculated with sterile PDA plugs. Three branches were inoculated with each isolate, and the experiment was repeated twice. The pathogenicity of the isolates was assessed 14 dpi. Re-isolations were made from all symptomatic cuttings using the same methods as for isolation.

### 2.6. Cultivar Susceptibility Testing

In order to assess the susceptibility of blueberry cultivars to infection, a selected *L. iraniensis* isolate (421-19) was used for the inoculations of branches of six-year-old healthy plants of nine different blueberry cultivars (‘Aurora’, ‘Barbara Ann’, ‘Bluecrop’, ‘Bluejay’, ‘Draper’, ‘Duke’, ‘Huron’, ‘Patriot’ and ‘Spartan’). The experiment was carried out as previously described for the pathogenicity testing. The disease intensity of the nine blueberry cultivars was assessed after 14 dpi. For the purpose of rating, the following 0–4 scale was established in this study based on symptom intensity: 0—no reaction; 1—surface necrosis near the wounded spot; 2—necrosis length from 2 to 20 mm; 3—necrosis length from 21 to 40 mm; and 4—necrosis length greater than 40 mm. The inoculations were performed in 3 replicates and the entire experiment was performed twice. The data were analyzed with the SPSS Software (version 29, IBM, USA) using one-way ANOVA followed by Duncan’s multiple range test at *p* < 0.05.

## 3. Results

### 3.1. Disease Symptoms and Isolates

**Figure 2:**
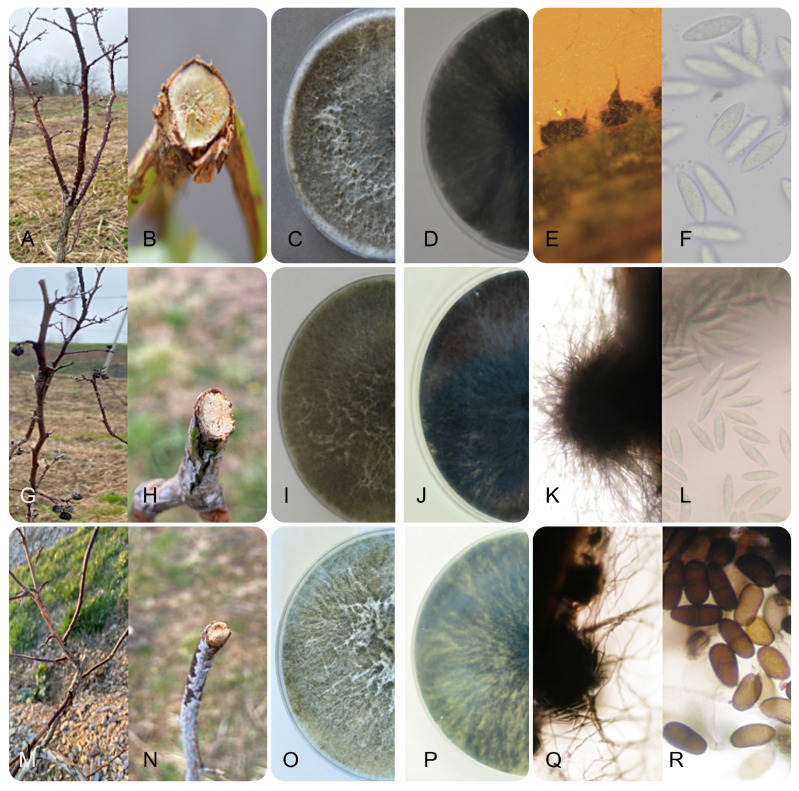
Symptomatology and morphology of Botryosphaeriaceae isolates from blueberry in Serbia: stem blight, wilting and inner tissue necrosis caused by *Neofusicoccum parvum* (**A**,**B**), *Botryosphaeria dothidea* (**G**,**H**) and *Diplodia seriata* (**M**,**N**); surface and reverse side of two weeks old colonies on PDA of *N. parvum* (**C**,**D**), *B. dothidea* (**I**,**J**) and *D. seriata* (**O**,**P**); pycnidium and conidia four weeks post inoculation on PNA of *N. parvum* (**E**,**F**), *B. dothidea* (**K**,**L**) and *D. seriata* (**Q**,**R**).

**Figure 3:**
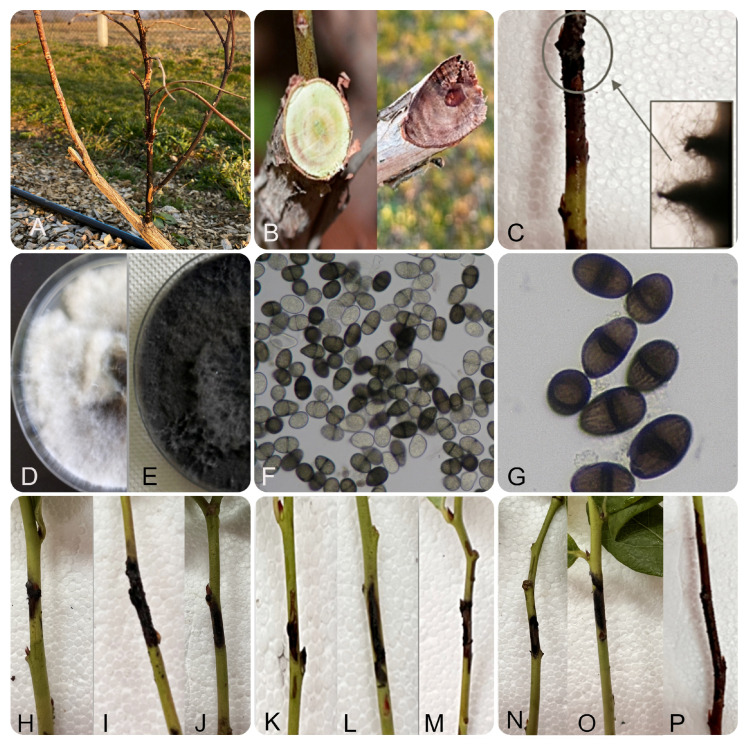
Symptomatology and morphology of *Lasiodiplodia iraniensis* isolates from Serbia: stem blight and wilting of blueberry (**A**); inner tissue necrosis (**B**); numerous pycnidia protruding bark on diseased branches (**C**); one (**D**) and two week old colonies on PDA (**E**); unicellular hyaline immature and pigmented mature conidia four weeks post inoculation on PNA (**F**); pigmented, 1-septate conidia with longitudinal striations (**G**), necrosis on inoculated branches of nine different blueberry cultivars: ‘Aurora’, ‘Spartan’, ‘Barbara Ann’, ‘Patriot’, ‘Huron’, ‘Draper’, ‘Bluejay’, ‘Bluecrop’ and ‘Duke’ (**H**–**P**).

During the survey, diseased blueberry plants were observed at the nine locations in Serbia (Figure 1), from which 153 samples were collected (Table 1), resulting in 236 isolates, of which representative monosporial isolates were morphologically categorized into 11 morphogroups, of which one or several representative isolates were identified by sequencing the ITS region to the genus level. A total of 128 Botryosphaeriaceae-like isolates were detected in single (three locations) or mixed infection with several non-Botryosphaeriaceae species (Table 1). Symptomatic plants were randomly distributed in groups along the rows or patches of different sizes in the orchards. All sampled orchards were up to six years old and disease incidence was estimated at sampling and ranged between 10 and 30% (mean 20.6%). The plants showed symptoms such as twig dieback, stem blight and wilt, followed by whole plant decay (Figure 2A,G,M and Figure 3A,C). Cross sections of symptomatic branches showed varying degrees of internal tissue necrosis, which correlated with symptom intensity (Figure 2B,H,N and Figure 3B). The spatial distribution of diseased plants along rows or groups of plants in close proximity is probably due to long-distance dispersal by planting material, which is responsible for the introduction of pathogens into the orchards, as well as the spread of the inoculum over short distances within the orchards by the movement of raindrops. Among the Botryosphaeriaceae-like isolates, four species were detected by ITS sequencing. *N. parvum* was the most prevalent with a detection frequency of 34.75% (82 isolates out of 236) (six out of nine localities). *B. dothidea* was detected in two localities with a detection frequency of 13.98% (33/236). *D. seriata* and *L. iraniensis* were both represented by five isolates from two individual localities with a detection frequency of 6.25 and 3.91, respectively. No species-specific symptomatology was observed. For further detailed characterization, twenty-four isolates were selected, eight isolates of *N. parvum*, seven of *B. dothidea,* four of *D. seriata* and five of *L. iraniensis*.

### 3.2. Fungal Morphology

The observed morphological characteristics of 70 Botryosphaeriaceae isolates from blueberries in Serbia showed stable uniform morphological characteristics within the four Botryosphaeriaceae species detected.

All 40 *N. parvum* isolates had a uniform appearance and initially formed white colonies with a grey centre after three days of incubation. With ageing, the colour of the colonies changed in all isolates, so that after seven days they were olive-grey on the surface and greenish grey on the reverse side and finally after two weeks of incubation they were dark grey on the surface and almost black on the reverse side. The aerial mycelium of all isolates was woolly and dense and often grouped in tufts that reached the lid of the Petri dish (Figure 2C,D). All isolates grew fast with average daily growth rates of 14.83 ± 1.09 mm, with no statistical differences between isolates. On PNA, all isolates formed globose blackish pycnidia after two weeks with average dimensions of (250-) 619.80 (-1250) × (200-) 547.75 (-1000) µm (Figure 2E), in which hyaline, fusiform to ellipsoidal, aseptate conidia (16.82–19.06 × 6.54–10.54 µm, average 17.94 ± 1.12 µm × 8.59 ± 2.05 µm) were visible after four weeks of incubation (Figure 2F).

All 20 isolates of *B. dothidea* also showed a uniform morphology and initially formed white, almost transparent colonies, which became darker in the centre after three days and dark olive-grey after seven days. With ageing after two weeks of incubation, the colonies became dark grey on the surface and dark brown, almost black, on the reverse, all with dense aerial mycelium, and often grouped in tufts that reached the lid of the Petri dish (Figure 2I,J). The average growth rate for all isolates was 13.5 ± 0.92 mm, with no statistical differences. After two weeks of incubation on PNA, all isolates developed blackish pycnidia with an average size of (230-) 365 (-500) × (200-) 287.5 (-375) µm (Figure 2K), with hyaline, fusiform, mostly aseptate conidia (24.91–29.09 × 6.88–9.12 µm, average 27 ± 2.09 × 8 ± 1.12 µm) developed well after four weeks of incubation (Figure 2L).

All five isolates of *D. seriata* also showed no differences in the appearance of the colonies and initially formed whitish colonies with a visible light olive-brown centre after three days. With further incubation, the colonies became dark olive-grey on the surface and dark grey, almost black, on the reverse after two weeks (Figure 2O,P). The aerial mycelium was dense and fluffy. The colonies of all isolates were fast growing with average growth of 26.2 ± 1.05 mm, overgrowing the entire surface of the Petri dish within two days, with no statistical differences. After two weeks of incubation on PNA, blackish pycnidia (Figure 2Q) could be observed, but they were immersed in the needles, woolly and densely covered with mycelium, making it difficult to determine the exact dimensions. One week after pycnidia formation (three weeks after inoculation on PNA), ovoid to oblong, elliptical, aseptate conidia could be observed (Figure 2R), which were initially hyaline and turned brown with age and were mainly uniseptate (22.95–26.75 × 9.50–10.75 µm, average 25 × 10 ± 1.15 μm), with no statistical differences between the five characterized isolates.

All five isolates of *L. iraniensis* exhibited uniform morphological characteristics on PDA and formed fast-growing, abundant aerial colonies with an average daily growth rate of 23.6 ± 1.12 mm and overgrew the surface of a 90 mm Petri dish in two days. Initially, the colonies of all isolates were whitish to smoky grey and became grey to olivaceous at the surface and dark, almost black, on the reverse side after two weeks (Figure 3D,E). Sporulation was induced on PNA and blueberry branches, where all isolates formed globose, black pycnidia covered with a dense mycelium (with average dimensions of (520-) 612.5 (-950) × (300-) 400 (-450) µm) after 14 dpi (Figure 3C). The presence of unicellular, hyaline, grey, immature conidia was recorded three weeks after inoculation (19.60–24.40 × 15.0 µm, average 22.00 ± 2.4 × 15.00 ± 0 µm). Approximately 60% of the conidia were pigmented, ellipsoid to ovoid, 1-septate with longitudinal striations (average (20.15–23.35 × 9.75–12.75 µm, 21.75 ± 1.6 × 11.25 ± 1.5 µm) four weeks after inoculation (Figure 3F), and after five weeks, all conidia were mature and pigmented (Figure 3G). There were no statistical differences between the five isolates in terms of growth rate and length of immature versus mature conidia (), except that the immature conidia were wider compared to the mature conidia (Fisher LSD method and 95% confidence).

Ecological characterization of *L. iraniensis* showed that none of the isolates were able to grow at 5 °C and 40 °C, while growth was recorded at cardinal temperatures of 10 and 37.5 °C (average daily growth for all isolates 6.9 and 2.65 mm, respectively). All isolates grew fastest at 25 and 35 °C (average for all isolates 23.6 and 22.35 mm, respectively). None of the isolates produced pink pigment on PDA at 35 °C in darkness.

### 3.3. Molecular Identification and Phylogenetic Analyses

BLASTn analyses for each of the ITS, *TEF1-α* and *TUB2* sequences of the morphologically characterized isolates confirmed the identification and proved that eight *N. parvum* isolates generated in this study share 98.5–100% nucleotide (nt) similarity with *N. parvum* ex-type isolate CBS 112931, seven isolates of *B. dothidea* 97.4–100% nt similarity with *B. dothidea* ex-type isolate CBS 115476 and four isolates of *D. seriata* 96.1–100% similarity with *D. seriata* ex-type (CBS 112555). The sequences of five *L. iraniensis* isolates had 100% nt similarity and 99.6–100% nt sequence similarity to sequences of *L. iraniensis* (including the ex-type isolate CBS 124710), *L. pseudotheobromae, L. theobromae* and *L. gonubiensis*. Similarly, *TEF1-α* and *TUB2 Lasiodiplodia* sequence analyses showed that the Serbian isolates have 99.3–99.7% and 97.2–100% nt sequence similarity with sequences of multiple *Lasiodiplodia* species, respectively.

**Figure 4:**
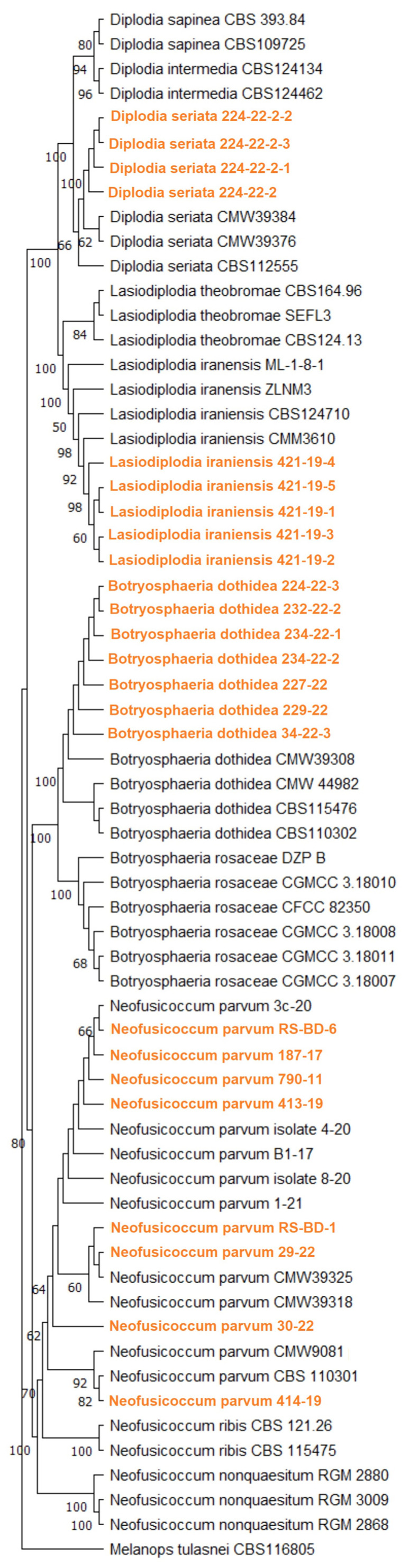
Maximum likelihood phylogenetic tree inferred from concatenated ITS rDNA, *TEF1-α* and *TUB2* genes of 24 Serbian and 10 previously listed type-derived Botryosphaeriaceae species (38 reference isolates) and *Melanops tulasnei* as an outgroup. Phylogram was generated with MEGA X using Tamura-Nei model Gamma distributed (G+I) [44]. Bootstrap analysis was performed with 1000 replicates and bootstrap values (>50%) are shown next to relevant branches. The Serbian Botryosphaeriaceae isolates are orange coloured.

A multi-locus phylogenetic analysis based on the combined ITS, *TEF1-α* and *TUB2* gene regions using the Maximum likelihood method which included 24 Serbian sequences from four species (*N. parvum*, *B. dothidea*, *D. seriata*, *L. iraniensis*) and 40 selected isolates from the Botryosphaeriaceae family belonging to 10 species yielded a phylogenetic tree that clearly resolved the topology of several well-supported clades corresponding to *N. parvum*, *B. dothidea*, *D. seriata*, *L. iraniensis* and other related species. The Serbian isolates clustered within their respective species clades and formed well-supported subclades together with the corresponding ex-type or reference strains (Figure 4). Within the *N. parvum* clade four subclades are indicated, all comprising 1–4 isolates from Serbia, demonstrating the diversity of blueberry isolates in Serbia.

**Figure 5:**
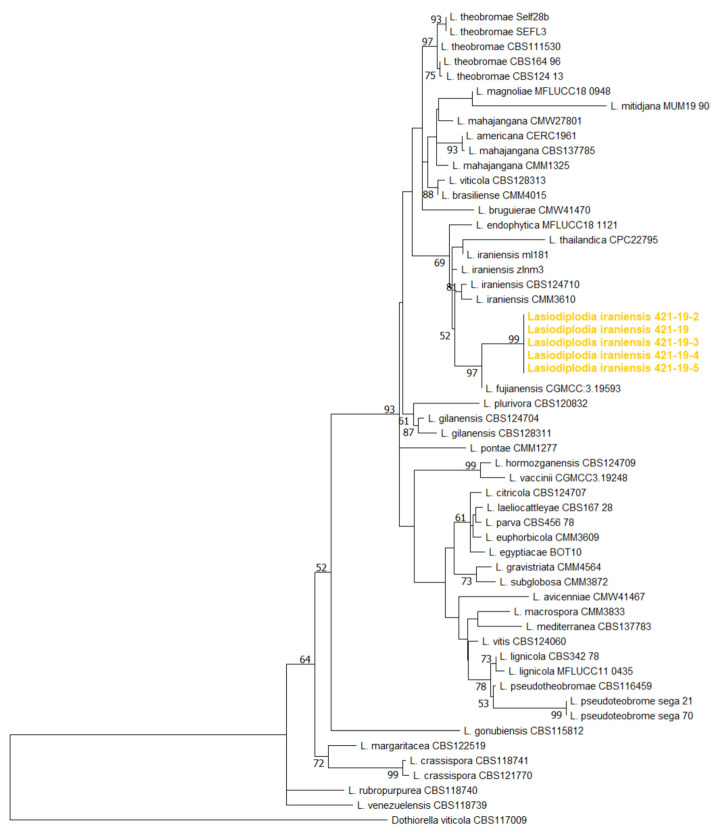
Maximum likelihood phylogenetic tree inferred from concatenated ITS rDNA, *TEF1-α* and *TUB2* genes of 5 Serbian and 34 previously listed type-derived species (54 reference isolates) *Lasiodiplodia* spp. and *Dothiorella viticola* as an outgroup. Phylogram was generated with MEGA X using Tamura-Nei model Gamma distributed (G+I) [44]. Bootstrap analysis was performed with 1000 replicates and bootstrap values (>70%) are shown next to relevant branches. The Serbian *Lasiodiplodia iraniensis* isolates are orange coloured.

Phylogenetic analyses of the ITS, *TEF1-α* and *TUB2* sequences using the Maximum likelihood method, which included five Serbian and fifty selected *Lasiodiplodia* isolates belonging to 35 species, yielded a phylogenetic tree whose topology and resolution are consistent with previous identification of publicly available isolates (Figure 5). The well-supported branch, which includes the closely related *L. iraniensis*, *L. fujianensis*, *L. thailandica* and *L. endophytica*, also included all Serbian isolates more closely related to *L. iraniensis* and *L. fujianensis*.

Subsequent *TEF1-α* sequence analyses of *L. iraniensis*, *L. fujianensis*, *L. thailandica* and *L. endophytica* revealed polymorphism at several positions (Table 3). In the analyzed set, *L. thailandica* isolates were unique as they had adenine at positions 14 and 55 and an insertion at positions 60–67, while all isolates of *L. iraniensis*, including Serbian isolates, were unique as they had adenine at positions 16 and 68. *L. iraniensis* could be easily distinguished from the closely related *L. fujianensis*, which is also a pathogen of blueberries [17] and has cytosine and thymine at positions 16 and 68, respectively. In addition, all 15 *L. iraniensis* isolates available to date form two separate haplotypes based on single nucleotide polymorphism, as they have either cytosine (9 isolates) or thymine (5 isolates) at position 137.

### 3.4. Pathogenicity

All 70 detected isolates of Botryosphaeriaceae caused visible symptoms 14 dpi on all inoculated branches, which completely resembled the symptoms of a natural infection. All isolates showed uniform pathogenicity and caused similar reactions in terms of the appearance and intensity of symptoms on the inoculated branches. A large number of pycnidia were observed in the necrotic area of all inoculated branches. No symptoms were observed on the control plants. All isolates were easily reisolated from all inoculated and symptomatic branches, so that Koch’s postulates were fulfilled.

### 3.5. Cultivar Susceptibility

**Figure 6:**
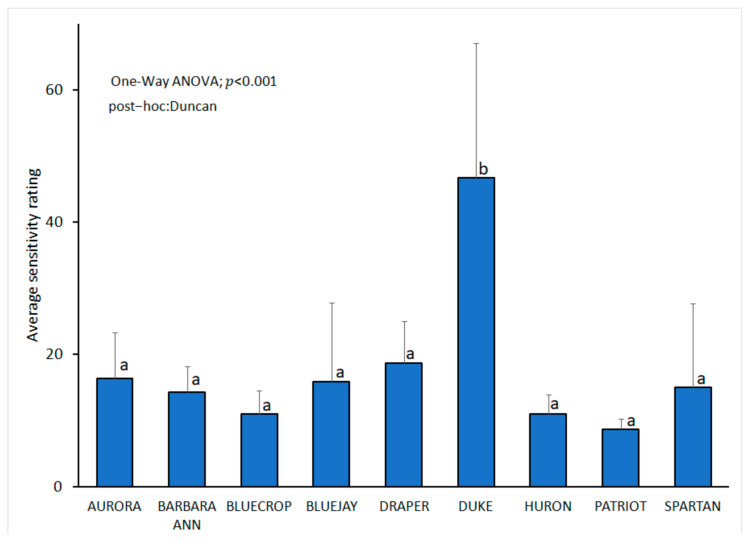
*Lasiodiplodia iraniensis*: susceptibility of nine blueberry cultivars analyzed with one-way ANOVA followed by Duncan’s multiple range tests at *p* < 0.05 using SPSS software (IBM, USA) assessed 14 days post inoculation, following 0–4 scale based on the symptom intensity: 0—no reaction; 1—surface necrosis near wounded spot; 2—necrosis lengths from 2 to 20 mm; 3—necrosis lengths from 21 to 40 mm, 4—necrosis length longer than 40 mm. The bars represent standard deviation. Values labelled with the same letter do not differ significantly.

When evaluating the response of cultivars to inoculation with the selected *L iraniensis* isolate 421-19, visible symptoms of necrosis were well developed 14 dpi on all inoculated branches of all nine blueberry cultivars tested, namely ‘Aurora’, ‘Barbara Ann’, ‘Bluecrop’, ‘Bluejay’, ‘Draper’, ‘Duke’, ‘Huron’, ‘Patriot’ and ‘Spartan’. The appearance of symptoms was similar in all cultivars and resembled a natural infection, while the control plants of all inoculated cultivars showed no symptoms. The intensity of necrosis varied from cultivar to cultivar (Figure 2H–P), and statistical analysis revealed that symptom development was significantly dependent on cultivar (*p* < 0.001). The cultivar ‘Duke’ proved to be the most susceptible cultivar with an average score of 3.17 ± 0.983, while the remaining eight cultivars formed a statistically uniform group with similar disease intensity, with average scores of 1.049 ± 0.105 (‘Aurora’)–2.17 ± 0.983 (‘Bluejay’ and ‘Draper’) (Figure 6).

## 4. Discussion

As a result of our symptom-based study of blueberry dieback in the main growing areas, we found *N. parvum*, *B. dothidea*, *D. seriata* and *L. iraniensis*, mainly in the form of mixed infections, causing stem blight and plant decay with an average disease incidence of over 20% in all orchards in Serbia. Both *D. seriata* and *L. iraniensis* were detected for the first time on blueberries in Serbia, and *L. iraniensis* was detected for the first time on blueberries worldwide.

*N. parvum* and *B. dothidea* are known to have a broad host range [21,52] and were recently detected on blueberries in Serbia [36]. Our study revealed a high prevalence of *N. parvum*, which is comparable to other blueberry growing areas in the world [8,38,54]. In Serbia, both *N. parvum* and *B. dothidea* are known pathogens of trees and shrubs [45,55,56,57], and in addition, *B. dothidea* is a known post-harvest pathogen of apples and quinces [58,59,60] and a root rot pathogen of sugar beet [47]. Although *D. seriata*, the third species detected, is known to infect trees and shrubs in Serbia [55] and after harvest on apples and quinces [59,61], it was not known to infect blueberries prior to our study. *D. seriata* is not very common in blueberries worldwide and has been found in a couple of samples in New Zealand [6,10] and the United States [62], which is similar to the situation in Serbia.

The fourth species detected, *L. iraniensis*, is a new pathogen for Serbia and was detected in blueberries for the first time worldwide. *L. iraniensis* has so far been detected mainly in tropical plants and nuts [19,20,22,23,24,25,26,27,28,29,30,31,32]. To date, at least 10 different *Lasiodiplodia* species have been described as blueberry pathogens. *L. chinensis* [16], *L. clavispora, L. fujianensis, L. henanica, L. nanpingensis* [17] and *L. pseudotheobromae* have been described in China [63], and *L. laeliocattleyae* in Peru [50]. *L. mediterranea* has been recorded in the USA [64], Australia [65] and Mexico [66], while *L. theobromae* occurs in Spain [9,67], China [8], Peru [50], the USA [7] and Australia [65]. *L. vaccinii* has been recorded in China [16], which shows that this genus could be associated with blueberries in general.

Infected blueberry plants in Serbia showed typical symptoms of Botryosphaeria stem blight [9,11,17,50] and could not be associated with any of the four detected species. Conventional identification of the Serbian isolates based on morphology and growth rate showed that they share characteristics of *N. parvum* [9,14,68,69,70,71,72], *B. dothidea* [69,72,73,74,75,76,77], *D. seriata* [59,61,72,78,79,80,81] and *L. iraniensis* [19,20,30,32]

Phylogenetic analyses of all detected Botryosphaeriaceae not only confirmed the identity of *N. parvum*, *B. dothidea* and *D. seriata*, but also revealed considerable diversity among isolates of *N. parvum* in Serbia, which is comparable to the high genetic variation observed in the New Zealand population from grapevines [82] or in Korea on Japanese bay trees [70] as well as in the production of pathogenicity-related toxins in *N. parvum* populations in France and Portugal [83]. A previous characterization of the population of *N. parvum* from blueberries in Serbia [36] did not reveal significant diversity among isolates, possibly due to a lower number of sampled orchards where infection could be due to a single introduction. In our study, diversity was shown to be the likely result of multiple introductions. The reverse situation and low diversity within the *B. dothidea* branch and between isolates from Serbia were similar to the situation of walnut in France [84] and olive in Croatia [72]. The low diversity among isolates of *D. seriata* detected in our study is to be expected as all isolates originated from a single field, probably as a result of a single introduction, although some variability in the *D. serata* population has been observed elsewhere [85,86].

The identity of Serbian *L. iraniensis* within the Botryosphaeriaceae as well as within *Lasiodiplodia* spp. could not be fully confirmed by phylogenetic analyses based on three loci, as has already been shown for some *Lasiodiplodia* isolates [52,62]. The Serbian isolates branched with the closely related *L. iraniensis*, *L. fujianensis*, *L. thailandica* and *L. endophytica* [17,21,49], but also share some of the morphological characteristics such as colony appearance and growth rate [17,19,20,30,32,87]. All four species can be clearly distinguished by the presence and size of the pycnidium as well as the septation and colour of the mature conidia. The Serbian *L. iraniensis* isolates form pycnidia with an average size of 612.5 µm (up to 850 µm), which is consistent with previously published values for *L. iraniensis* (up to 980 µm, [20]) and differs markedly from the larger pycnidia of *L. fujianensis* (up to 1.3 mm, [17]) and the much smaller pycnidia of *L. thailandica* [87], while *L. endophytica* does not sporulate in culture [49]. The morphology of the conidia is also a solid tool to distinguish between these four species. Serbian and all previously published isolates of *L. iraniensis* form pigmented, dark brown, mature conidia that are 1-septate [19,20,31,32,88,89]. The closely related *L. fujianensis* can be easily distinguished as it forms pigmented but aseptate mature conidia [17], while *L. thailandica* is characterized by the fact that most mature conidia remain hyaline [49,87,88].

Sequence analysis of the *TEF1-α* gene provided further confirmation of the clear distinction of *L. iraniensis* from the closely related *L. fujianensis*, which was recently detected as a blueberry pathogen in China [17], and from the phylogenetically closely related *L. thailandica* and *L. endophytica*, which were not recorded as blueberry pathogens. All available *L. iraniensis*, including the five Serbian isolates, shared adenine at positions 16 and 68 of the analyzed fragment of the *TEF1-α* gene, which is a unique sequence feature. In our study, we also found that the previously characterized population of *L. iraniensis* consists of two haplotypes based on the presence of cytosine or thymine at position 137 of the *TEF1-α* gene, which represents the first worldwide population analysis of this pathogen. The Serbian isolates belong to the rarer cytosine–haplotype and are identical to the *L. iraniensis* isolates from *Jatropha curcas* in Brazil (described as *L. jatrophicola*, [17,53]) and sweet orange in the USA [32]. The potential role and importance of this diversity in the *L. iraniensis* population will likely become clearer as additional data and isolates become available and characterized.

There are no studies on the susceptibility of different blueberry accessions or cultivars to *Lasiodiplodia* spp. Even the data on cultivars naturally infected with *Lasiodiplodia* spp. are limited. In China, *L. theobromae* was isolated from the cultivar ‘Misty’ and *L. pseudotheobromae* from M6 [8]. Our studies on the susceptibility of nine blueberry cultivars are valuable and provide the first data on the presence of different levels of susceptibility in nine tested cultivars. Blueberry ‘Duke’ was found to be significantly more susceptible compared to the other cultivars, which should be further confirmed under different conditions and in other blueberry growing regions. The tested cultivars ‘Aurora’, ‘Bluecrop’ and ‘Bluejay’, which are predominant in blueberry cultivation in the USA [90], responded well and developed low disease severity. The observed difference between the blueberry cultivars tested and the fact that *L. iraniensis* was isolated from ‘Duke’ in this study may indicate a possible link between natural infection and susceptibility of a particular cultivar.

In Serbia, blueberry ‘Duke’ as the most commonly grown cultivar [3,4], characterized by high susceptibility, is seriously threatened by Bortyosphaeriaceae and especially the emergence of *D. seriata* and *L. iraniensis* as new blueberry pathogens. Limiting options for the overall management of Botryosphaeriaceae stem blight diseases emphasize the use of disease-free planting material and the avoidance of injuring plants [11]. In our study, the majority of orchards were in their second or third year of production, meaning that planting material is a likely source of infection, as has been shown previously for many Botryosphaeriaceae [10,21,91]. It would be beneficial for Serbian producers if the control of production and, above all, the import of blueberry planting material in Serbia were strengthened and improved. In view of the fact that the quarantine status of *L. pseudotheobromae* and *L. iraniensis* has been discussed [18,19], the standard procedure in the international trade of blueberry planting material should be analyzed and reconsidered. Our results offered a solution as we identified less or moderately susceptible blueberry cultivars to be grown in the affected areas and even more emphasized the need to use pathogen-free planting material in all blueberry-growing areas worldwide.

## Figures and Tables

**Table 1:** Geographic distribution of isolates collected in Serbia.

No. *	Year	District	Locality	Blueberry Cultivar	Estimated Disease Incidence (%)	No. of Collected Samples	Fungal Species Detected (Positive Samples)	Isolates	
								Culture	ITS Seq
1	2011	Kolubara	Belanovica	Duke	20	20	*Neofusicoccum parvum* (16) *Neopestalotiopsis* spp. (11) *Botrytis* spp. (5) *Epicoccum* spp. (7)	8	1
								4	1
								3	1
								1	1
2	2017	Srem	Šid	Duke	20	20	*Neofusicoccum parvum* (18) *Neopestalotiopsis* spp. (14)	14 11	2 1
3	2019	Srem	Irig	Duke	15	5	*Lasiodiplodia iraniensis* (5)	5	5
4		Kolubara	Ub	Duke	20	25	*Neofusicoccum parvum* (18)	6	2
5	2020	Belgrade	Sopot	Duke	10	8	*Neofusicoccum parvum* (7) *Alternaria* spp. (4)	4	1
6		Moravica	Gornji Milanovac	Duke	30	7	*Neofusicoccum parvum* (5)	4	1
7	2022	Belgrade	Slatina	Duke	15	20	*Neofusicoccum parvum* (18) *Diaporthe* spp. (12) *Peroneutypa* spp. (7) *Fusarium* spp. (10)	4 8 2 2	1 1 1 2
8		Kolubara	Jajčić	Duke	30	25	*Botryosphaeria dothidea* (18) *Diplodia seriata* (8) *Diaporthe* spp. (5) *Neopestalotiopsis* spp. (16)	12 5 1 4	4 4 1 3
9		Kolubara	Slavkovica	Duke	25	23	*Botryosphaeria dothidea* (15) *Diaporthe* spp. (17)	8 8	3 2


* Serial number of the locality as indicated on the map (Figure 1).

**Table 2:** Isolates of the *Botryosphaeriaceae* species used in this study.

GenBank Accessions						
Species	Strain/Isolate	Host	Country	ITS	*TEF1-α*	*β-Tubulin*
*Botryosphaeria dothidea*	CBS115476	*Prunus* sp.	Switzerland	AY236949	AY236898	AY236927
*Botryosphaeria dothidea*	CBS110302	*Vitis vinifera*	Portugal	AY259092	AY573218	EU673106
*Botryosphaeria dothidea*	CMW44982	*Sequoiadendron* *giganteum*	Serbia	KF575008	KF575040	KF575104
*Botryosphaeria dothidea*	CMW39308	*Sequoiadendron* *giganteum*	Serbia	KF575008	KF575040	KF575104
***Botryosphaeria dothidea***	**34-22-3**	***Vaccinium corymbosum***	**Serbia**	**PV235336**	**PV296171**	**PV278143**
***Botryosphaeria dothidea***	**234-22-1**	***Vaccinium corymbosum***	**Serbia**	**PV268085**	**PX056801**	**PX056807**
***Botryosphaeria dothidea***	**227-22**	***Vaccinium corymbosum***	**Serbia**	**PV263064**	**PX056800**	**PX056806**
***Botryosphaeria dothidea***	**229-22**	***Vaccinium corymbosum***	**Serbia**	**PV268086**	**PX056799**	**PX056805**
***Botryosphaeria dothidea***	**224-22-3**	***Vaccinium corymbosum***	**Serbia**	**PV263065**	**PX056804**	**PX056810**
***Botryosphaeria dothidea***	**234-22-2**	***Vaccinium corymbosum***	**Serbia**	**PV263170**	**PX056802**	**PX056808**
***Botryosphaeria dothidea***	**232-22-2**	***Vaccinium corymbosum***	**Serbia**	**PX048943**	**PX056803**	**PX056809**
*Botryosphaeria rosaceae*	CBSCGMCC 3.18007	*Malus* sp.	China	KX197074	KX197094	KX197101
*Botryosphaeria rosaceae*	CBSCGMCC 3.18008	*Amygdalus* sp.	China	KX197075	KX197095	KX197102
*Botryosphaeria rosaceae*	CFCC 82350	*Malus* sp.	China	KX197079	KX197097	KX197106
*Botryosphaeria rosaceae*	CGMCC3.18009	*Malus* sp.	China	KX197076	KX197096	KX197103
*Botryosphaeria rosaceae*	CBSCGMCC 3.18010	*Pyrus* sp.	China	KX197077	-	KX197104
*Botryosphaeria rosaceae*	CBSCGMCC 3.18011	*Pyrus* sp.	China	KX197078	-	KX197105
*Diplodia intemerdia*	CBS124134	*Cydonia* sp.	Portugal	HM036528	GQ923851	KX464798
*Diplodia intermedia*	CBS124462	*Malus sylvestris*	Portugal	GQ923858	GQ923826	-
*Diplodia sapinea*	CBS393.84	*Pinus nigra*	Netherlands	DQ458895	DQ458880	DQ458863
*Diplodia sapinea*	CBS109725	*Pinus patula*	South Africa	DQ458896	DQ458881	DQ458864
*Diplodia seriata*	CMW39384	*Thuja occidentalis*	Serbia	DQ458896	DQ458881	DQ458864
*Diplodia seriata*	CMW39376	*Chamaecyparis* *pisifera*	Serbia	KF574996	KF575027	KF575092
*Diplodia seriata*	CBS112555	*Vitis vinifera*	Portugal	AY259094	AY573220	DQ458856
***Diplodia seriata***	**224-22-2**	***Vaccinium corymbosum***	**Serbia**	**PV263172**	**PV296172**	**PV278144**
***Diplodia seriata***	**224-22-2-1**	***Vaccinium corymbosum***	**Serbia**	**PX023087**	**PX056793**	**PX056796**
***Diplodia seriata***	**224-22-2-2**	***Vaccinium corymbosum***	**Serbia**	**PX022810**	**PX056794**	**PX056797**
***Diplodia seriata***	**224-22-2-3**	***Vaccinium corymbosum***	**Serbia**	**PX022815**	**PX056795**	**PX056798**
*Dothiorella viticola*	CBS 117009	*Vitis vinifera*	Spain	AY905554	AY905559	EU673104
*Lasiodiplodia americana*	CERC 1961	*Pistacia vera*	USA: Arizona	KP217059	KP217067	KP217075
*L. avicenniae*	CMW 41467	*Avicennia marina*	South Africa	KP860835	KP860680	KP860758
*L. brasiliense*	CMM 4015	*Mangifera indica*	Brazil	JX464063	JX464049	-
*L. bruguierae*	CMW 41470	*Bruguiera gymnorrhiza*	South Africa	NR_147358	KP860678	KP860756
*L. citricola*	CBS 124707	*Citrus* sp.	Iran	GU945354	GU945340	KP872405
*L. crassispora*	CBS 118741	*Santalum album*	Australia (WA)	DQ103550	EU673303	EU673133
*L. crassispora*	CBS 121770	*Acacia mellifera*	Namibia	EU101307	EU101352	-
*L. endophytica*	MFLUCC 18-1	*Magnolia candolii*	China	MK501838	MK584572	MK550606
*L. egyptiacae*	CBS 130992	*Mangifera indica*	Egypt	JN814397	JN814424	-
*L. euphorbicola*	CMM 3609	*Jatropha curcas*	Brazil	KF234543	KF226689	KF254926
*L. fujianensis*	CGMCC: 3.19593	*Vaccinium corymbosum*	China	MK802164	OM144905	MK816337
*L. gilanensis*	CBS 124704	*Citrus* sp.	Iran	GU945351	GU945342	KP872411
*L. gilanensis*	CBS 128311	*Vitis vinifera*	USA: Missouri	HQ288225	HQ288267	-
*L. gonubiensis*	CBS 115812	*Syzygium cordatum*	South Africa	AY639595	DQ103566	DQ458860
*L. gravistriata*	CMM 4564	*Anacardium humile*	Brazil	KT250949	KT250950	-
*L. hormozganensis*	CBS 124709	*Olea* sp.	Iran	GU945355	GU945343	KP872413
*L. iraniensis*	ZLNM3	*Mangifera indica*	Taiwan	OR534158	OR552386	OR551924
*L. iraniensis*	ML-1-8-1	*Mangifera indica*	Taiwan	OR534131	OR552266	OR551897
*L. iraniensis*	CBS 124710	*Salvadora persica*	Iran	GU945346	GU945334	KP872415
*L. iraniensis*	CMM 3610	*Jatropha curcas*	Brazil	KF234544	KF226690	KF254927
***L. iraniensis***	**421-19-5**	***Vaccinium corymbosum***	**Serbia**	**OR856066**	**PP238619**	**PP238615**
***L. iraniensis***	**421-19-4**	***Vaccinium corymbosum***	**Serbia**	**OR878143**	**PP372561**	**PP238614**
***L. iraniensis***	**421-19-3**	***Vaccinium corymbosum***	**Serbia**	**OR856065**	**PP238618**	**PP238613**
***L. iraniensis***	**421-19-2**	***Vaccinium corymbosum***	**Serbia**	**OR856064**	**PP238617**	**PP238612**
***L. iraniensis***	**421-19**	***Vaccinium corymbosum***	**Serbia**	**OR727299**	**PP238616**	**PP238611**
*L. laeliocattleyae*	CBS 167.28	*Laeliocattleya* sp.	Italy	KU507487	KU507454	…
*L. lignicola*	MFLUCC 11-0435	On dead wood	Thailand	JX646797	KU887003	JX646845
*L. lignicola*	CBS 342.78	*Sterculia oblonga*	Germany	KX464140	KX464634	KX464908
*L. macrospora*	CMM 3833	*Jatropha curcas*	Brazil	KF234557	KF226718	KF254941
*L. magnoliae*	MFLUCC 18-0948	*Magnolia candolii*	China	MK499387	MK568537	MK521587
*L. mahajangana*	CBS 124927	*Terminalia catappa*	Madagascar	FJ900595	FJ900641	FJ900630
*L. mahajangana*	CMM 1325	*Citrus sinensis*	Brazil	KT154760	KT008006	KT154767
*L. mahajangana*	CBS 137785	*Retama raetam*	Tunisia	KJ638317	KJ638336	-
*L. margaritacea*	CBS 122519	*Adansonia gibbosa*	Australia (WA)	EU144050	EU144065	KX464903
*L. mediterranea*	CBS 137783	*Quercus ilex*	Italy	KJ638312	KJ638331	-
*L. mitidjana*	MUM 19.90	*Citrus sinensis*	Algeria: Mitidja	MN104115	MN159114	-
*L. parva*	CBS 456.78	*Manihot esculenta*	Colombia	EF622083	EF622063	KP872419
*L. plurivora*	CBS 120832	*Prunus salicina*	South Africa	EF445362	EF445395	KP872421
*L. pontae*	CMM 1277	*Spondias purpurea*	Brazil	KT151794	KT151791	KT151797
*L. pseudotheobromae*	CBS 116459	*Gmelina arborea*	Costa Rica	EF622077	EF622057	EU673111
*L. pseudotheobromae*	SEGA21	*Vaccinium corymbosum*	USA	JN607093	JN607116	JN607140
*L. pseudotheobromae*	SEGA70	*Vaccinium corymbosum*	USA	JN607095	JN607118	JN607142
*L. rubropurpurea*	CBS 118740	*Eucalyptus grandis*	Australia	DQ103553	EU673304	EU673136
*L. subglobosa*	CMM 3872	*Jatropha curcas*	Brazil	KF234558	KF226721	KF254942
*L. thailandica*	CBS 138760	*Mangifera indica*	Thailand	KJ193637	KJ193681	-
*L. theobromae*	CBS 111530	*Leucospermum* sp.	USA: Hawaii	EF622074	EF622054	-
*L. theobromae*	CBS 124.13	-	USA	DQ458890	DQ458875	DQ458858
*L. theobromae*	CBS 164.96	Fruit along coral reef coast	Papua New Guinea	AY640255	AY640258	EU673110
*L. theobromae*	SEFL3	*Vaccinium corymbosum*	USA	JN607091	JN607114	JN607138
*L. theobromae*	SEFL28b	*Vaccinium corymbosum*	USA	JN607092	JN607115	JN607139
*L. vaccinii*	CGMCC 3.19248	*Vaccinium corymbosum*	China	MK157131	MK157158	MK157149
*L. venezuelensis*	CBS 118739	*Acacia mangium*	Venezuela	DQ103547	EU673305	EU673129
*L. viticola*	CBS 128313	*Vitis vinifera*	USA: Arkansas	HQ288227	HQ288269	HQ288306
*L. vitis*	CBS 124060	*Vitis vinifera*	-	KX464148	KX464642	KX464917
*Melanops tulasnei*	CBS116805	*Quercus robur*	Germany	FJ824769	KF766423	FJ824780
*Neofusicoccum nonquaesitum*	RGM2880	*Vaccinium corymbosum*	Chile	MT790243	MT845319	MT832803
*Neofusicoccum nonquaesitum*	RGM3009	*Vaccinium corymbosum*	Chile	MT790223	MT845299	MT832783
*Neofusicoccum nonquaesitum*	RGM2868	*Vaccinium corymbosum*	Chile	MT790266	MT845342	MT832826
*Neofusicoccum parvum*	8-20	*Vaccinium corymbosum*	Serbia	OQ31660	OQ342772	OQ473020
*Neofusicoccum parvum*	3c-20	*Vaccinium corymbosum*	Serbia	OQ316605	OQ473018	OQ342770
*Neofusicoccum parvum*	B1-17	*Vaccinium corymbosum*	Serbia	OQ316604	OQ342769	OQ473017
*Neofusicoccum parvum*	4-20	*Vaccinium corymbosum*	Serbia	OQ316606	OQ342771	OQ473019
*Neofusicoccum parvum*	1-21	*Vaccinium corymbosum*	Serbia	OQ316608	OQ342773	OQ473021
*Neofusicoccum parvum*	CMW39325	*Aesculus* *hippocastanum*	Serbia	KF575021	KF575045	KF575117
*Neofusicoccum parvum*	CMW39318	*Chamaecyparis* *lawsoniana*	Serbia	KF575022	KF575046	KF575118
*Neofusicoccum parvum*	CBS110301	*Vitis vinifera*	Portugal	AY259098	AY573221	EU673095
*Neofusicoccum parvum*	ATCC58191 (CMW9081)	*Populus nigra*	New Zealand	AY236943	AY236888	AY236917
***Neofusicoccum parvum***	**413-19**	***Vaccinium corymbosum***	**Serbia**	**MW624690**	**OL456720**	**OL456719**
***Neofusicoccum parvum***	**414-19**	***Vaccinium corymbosum***	**Serbia**	**MW624691**	**OL456721**	**OL415487**
***Neofusicoccum parvum***	**790-11**	***Vaccinium corymbosum***	**Serbia**	**PV235269**	**PV278148**	**PV278140**
***Neofusicoccum parvum***	**187-17**	***Vaccinium corymbosum***	**Serbia**	**PV226107**	**PV278145**	**PV278139**
***Neofusicoccum parvum***	**29-22**	***Vaccinium corymbosum***	**Serbia**	**PV235282**	**PV278146**	**PV278137**
***Neofusicoccum parvum***	**30-22**	***Vaccinium corymbosum***	**Serbia**	**PV235306**	**PV278147**	**PV278138**
***Neofusicoccum parvum***	**RS-BD-1**	***Vaccinium corymbosum***	**Serbia**	**PV235313**	**PV278149**	**PV278141**
***Neofusicoccum parvum***	**RS-BD-6**	***Vaccinium corymbosum***	**Serbia**	**PV235322**	**PV278150**	**PV278142**
*Neofusicoccum ribis*	CBS121.26	*Ribes* sp.	USA	AF241177	AY236879	AY236908
*Neofusicoccum ribis*	CBS115475	*Ribes* sp.	USA	AY236935	AY236877	AY236906

The isolates in bold are obtained in this study.

**Table 3:** Translation elongation factor 1α gene nucleotide polymorphism of all available isolates of *Lasiodiplodia iraniensis,* *L. fujianensis*, *L. thailandica* and *L. endophytica*.

*Lasiodiplodia* spp. and Accession Number of the Isolate	Nucleotide Alignment Using *L. endophytica* MK584572 as Representative							
	14	16	55	60–67	68	128	137	159
*L.endophytica* MK584572 [49]	C	C	G	-	T	C	C	G
*L. thailandica* MW183805 [51]	A	C	A	insertion	T	T	C	G
*L. thailandica* OQ509100 [52]	A	C	A	insertion	T	C	C	G
*L. thailandica* KJ93681 [52]	A	C	A	insertion	T	T	C	G
*L. fujianensis* MK887178 [17]	C	C	G	-	T	C	C	C
*L. iraniensis* GU945334 [20]	C	A	G	-	A	C	T	C
*L. iraniensis* GU945336 [20]	C	A	G	-	A	C	T	C
*L. iraniensis* GU945337 [20]	C	A	G	-	A	C	T	C
*L. iraniensis* ON975017 [31]	C	A	G	-	A	C	T	C
*L. iraniensis* OR114284 [30]	C	A	G	-	A	C	T	C
*L. iraniensis* PP389268 [32]	-	-	G	-	A	C	C	C
*L. iraniensis* PP389275 [32]	-	-	G	-	A	C	C	C
*L. iraniensis* PP389256 [32]	-	-	G	-	A	C	C	C
*L. iraniensis* (syn. *L. jatrophicola*) KF226690 [53]	C	A	G	-	A	C	C	C
*L. iraniensis* PP238619 (this study)	C	A	G	-	A	C	C	C
*L. iraniensis* PP372561 (this study)	C	A	G	-	A	C	C	C
*L. iraniensis* PP238618 (this study)	C	A	G	-	A	C	C	C
*L. iraniensis* PP238617 (this study)	C	A	G	-	A	C	C	C
*L. iraniensis* PP238616 (this study)	C	A	G	-	A	C	C	C

Different background color indicates differences in nucleotides at particular position.

## Data Availability

Data set available on request from the authors.

## Abbreviations

The following abbreviations are used in this manuscript:

PDA	Potato Dextrose Agar
PNA	Pine Needle Agar
PDB	Potato Dextrose Broth
ITS	Internal Transcribed Spacer
TEF1-α	Translation Elongation Factor 1α
TUB2	Beta Tubulin
DNA	Deoxyribonucleic Acid
Nt	Nucleotide
Bp	Base Pair
Dpi	Days Per Inoculation
Cv	Cultivar
PCR	Polymerase Chain Reaction
